# Contaminated by Its Prior Use: Strategies to Design and Market Refurbished Personal Care Products

**DOI:** 10.1007/s43615-022-00197-3

**Published:** 2022-07-30

**Authors:** Theresa S. Wallner, Senna Snel, Lise Magnier, Ruth Mugge

**Affiliations:** Faculty of Industrial Design Engineering, Department of Design, Organization and Strategy, Delft University of Technology, Landbergstraat 15, Delft, 2628CE the Netherlands

**Keywords:** Circular economy, Refurbishment, Personal care products, Contamination, Product appearance, Sustainable consumption

## Abstract

Refurbishment is an effective circular strategy to lengthen a product’s lifetime. However, refurbished products that are intimately used, such as personal care products, cause a feeling of unease in consumers because they are perceived to be contaminated. In 15 in-depth online interviews with female users of intense pulsed light (IPL) device living in the Netherlands, we explored why consumers have contamination concerns regarding an IPL device and proposed strategies to decrease these. Participants felt that refurbished personal care products with signs of wear-and-tear were a riskier choice and expected that the device would malfunction, have a shorter product lifetime, and would be contaminated due to the previous use. Based on the location and amount of wear-and-tear, participants made inferences on how the prior user had treated the device. While light wear-and-tear indicated normal use, heavy wear-and-tear was interpreted as a sign of bad treatment by the previous user. To keep refurbished personal care products at their highest value, we suggest five design strategies to minimize contamination concerns by designing a product that smells and looks hygienic after multiple lifecycles: (1) using colors that evoke associations with hygiene, (2) making signs of wear-and-tear less visible, (3) using smooth (cleanable) materials, (4) minimizing the number of split lines in the product, and (5) giving refurbished products a clean product smell. For refurbished personal care products with signs of wear-and-tear that cannot be eliminated, we propose mitigating consumers’ contamination concerns with marketing strategies, such as fostering a good brand image, offering refurbished products at a lower price, with an extended warranty, and underlining expert check-ups and standards during refurbishment.

**Graphical abstract:**

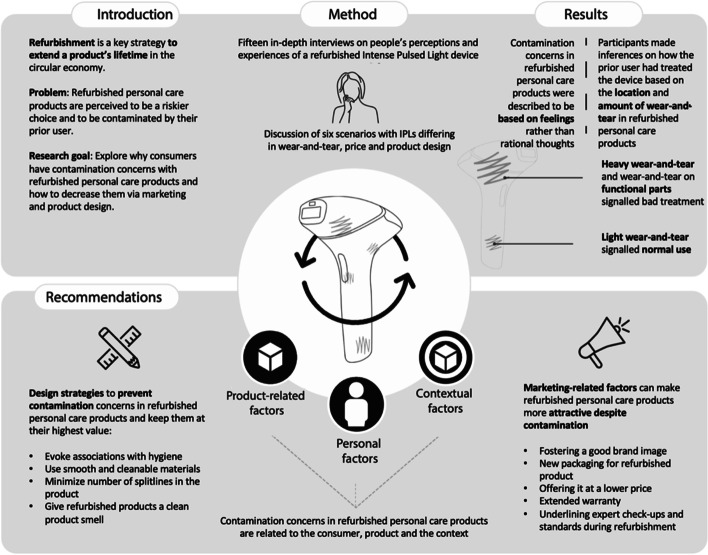

## Introduction

The average carbon footprint of a person living in the EU equaled to 6.7 tons of CO_2_ per year in 2019 [1]. One way of reducing a person’s carbon footprint is to design products that are used longer. By slowing loops, fewer products need to be produced, discarded, and incinerated. Consequently, the amount of virgin materials needed to manufacture products and inherent CO_2_ emissions is reduced, and electronic waste can be prevented [2, 3]. Refurbishment is a powerful strategy to extend a product’s lifetime in the circular economy. Based on Pigosso et al. [4], we define refurbished products as products that are collected after being used by a previous owner, tested, cleaned, and restored into an acceptable state and subsequently resold [4]. By doing this, products can be kept in the loop and therefore have a lower environmental impact compared to new products [3, 4]. Consumer electronics’ carbon footprint can be reduced by 87% if they are refurbished and resold instead of manufacturing new ones [5]. This, however, requires that products are designed to last multiple lifecycles and remain desirable to consumers [6]. Yet, consumers find refurbished products less desirable because they fear that refurbished products are contaminated by their prior use. Consumers’ contamination concerns with refurbished products can be described as feelings of unease or even disgust that consumers experience due to the prior use of the product [7, 8]. Especially, refurbished products that are used intimately, such as personal care products, are undesirable to consumers because they trigger more contamination concerns [9–11] and these contamination concerns have shown to be the largest predictor of why consumers choose new over refurbished products [12, 13]. While most research on refurbishment has focused on products that are less sensitive to contamination, such as smartphones [14–18], we explore personal care products that are intimately used and thereby trigger more contamination concerns [11]. The importance of our research, however, goes beyond refurbishment; contamination has shown to be a barrier in the consumption of various circular products [19–23] such as reused clothing [19, 24], products circulating in product service systems [25], remanufactured consumer electronics [9], or reusable food packaging [26, 27], and is therefore important to investigate. The aim of this paper is to uncover the underlying mechanisms triggering contamination concerns with refurbished products. This entails exploring how aspects related to the product design and marketing of refurbished personal care products trigger these feelings of unease. Furthermore, we aim to explore how different product design and marketing strategies can be used to decrease contamination concerns. With this research, we hence contribute to literature on user-centric design and marketing of refurbished products. Furthermore, we add to the literature on how to enhance the desirability of refurbished personal care products by mitigating contamination concerns.

### The Risks and Benefits Balance of Refurbished Products

Consumer decision-making for or against refurbished products is described as a tradeoff between risks and benefits. Consumers may be afraid of making a bad financial investment (financial risk) buying refurbished products that may not work satisfactorily (functionality risk) and need to be sent back, costing time (time risk; [7]). Additionally, they may worry that refurbished products have lower performance or fewer features compared to newer models (obsolescence risk; [15, 18]) or are simply not as durable as new products [12]. These risks are then weighted against the perceived benefits of refurbished products. A lower price and environmental benefits can incentivize consumers to buy refurbished products [10, 15, 17, 28–30]. A wide distribution of the refurbished product on the market, a good seller’s reputation [17], extended warranties [15], and marketing strategies, such as probabilistic selling [31], can decrease the perceived risks associated with refurbished smartphones. Greater perceived benefits and decreased perceived risks will positively influence purchase intentions of refurbished products [32].

Sociocultural factors [33] and consumer characteristics [13, 15, 34, 35] determine how consumers react to refurbished products, which in turn can help formulate strategies to market them. For example, Gaur et al. [33] argued that sociocultural factors, such as a harmony orientation towards nature, predict consumers’ purchase intentions of refurbished products. For consumers who are environmentally concerned, eco-labeling [17, 29, 36] and emphasis on green transportation [37] have shown to improve consumers’ purchase intentions of refurbished products.

Current marketing strategies of refurbished products, such as offering them at a lower price and with a warranty, have one shortcoming: they treat the consumer as a rational decision maker. While they make refurbished products a better rational tradeoff between risks and benefits, they ignore emotional factors that might be at play in consumers’ choices. Consumers have lower purchase intentions for refurbished products because they look used, and therefore feel contaminated as a result of their prior use [7, 38]. Consumers’ contamination concerns have shown to determine the product choice between new and refurbished products [12]. To design products that last multiple life cycles, consumers’ contamination concerns need to be taken into consideration when designing and marketing refurbished products.

### Consumers’ Contamination Concerns

Perceived contamination is described as a feeling of discomfort or disgust that arises when consumers interact with a reused product that is believed to be contaminated by its previous use [21]. These traces can be of physical nature, such as skin residue or oils, but can also be non-physical, such as data-traces in refurbished smartphones [7, 8].

Contamination is described to be of hygienic, utilitarian, or of territorial nature (HUT model, [38]). Hygienic contamination is triggered by the concern that an object poses a threat to one’s health because it may be contaminated with dirt or pathogens (e.g., bacteria cultivate on a device due to its material). Territorial contamination transpires when a previous user’s signs of use interfere with one’s personal space and therefore create a feeling of unease. One example would be a product that smells like a previous user’s perfume. Utilitarian contamination occurs when a product’s functionality is believed to be decreased (e.g., lower battery capacity in refurbished smartphones). On the one hand, concerns about the decreased functionality of refurbished products do not seem unfounded, as refurbished products are brought into an acceptable state and can have a lower performance. Whether the contamination of reused products, however, causes an uneasy feeling depends on the product itself and in which context it circulates [8]. While most consumers would be comfortable getting a shave at a barbers’ shop, buying a second-hand shaver would feel uncomfortable. This shows that contamination is highly dependent on the context and does not follow rational reasoning but is based on consumers’ feelings.

### The Influence of Contamination-Reducing Strategies

Strategies to reduce contamination can be related to the context of the product or the product itself [38] and have shown to influence the choice for refurbished products [13, 17, 32]. Consumers’ concern that a refurbished product has a lower functionality due to its prior use (utility contamination) and concerns about its hygiene (hygienic contamination) can be addressed by communicating about the product’s state. The refurbishment procedure entails that products are thoroughly cleaned during the refurbishment process and brought into an acceptable state. This is, however, not always clear to consumers [7]. Prior research therefore tested whether communicating that the product is cleaned and giving it a sparkling clean label would influence the choice for refurbished products by reducing its contamination [13]. This cleaning label was, however, less important in determining the product choice than other contamination-reducing strategies that decrease contamination via product design. The importance of a certified clean label could have been reduced because it unintendedly triggers users’ contamination concerns. In reused clothing, communicating that a product was cleaned and sterilized unintendedly reminded users of the product’s previous use [39]. Additionally, while a certified clean label might address consumers’ contamination concerns via the product’s marketing, it does not help to keep the product at its highest economic and environmental value. We will therefore explore how the product appearance can be optimized to prevent contamination concerns.

The product’s appearance helps consumers assess the quality and functionality of a product [40, 41]. Research on the contamination of circular products has shown that the product state (e.g., the degree to which the product is clean or unclean) and the product characteristics (e.g., visibility of wear-and-tear) can evoke associations of contamination [38]. Research on refurbished headphones has shown that most consumers choose refurbished products that show no signs of aesthetic wear-and-tear (e.g., scratches) and that have parts touching the skin (e.g., ear-cushions in headphones) renewed during the refurbishment process [13]. Renewing contamination-sensitive parts and preventing or eliminating signs of wear-and-tear during the refurbishment process were therefore suggested to be an effective strategy to enhance the desirability of refurbished products. Furthermore, while consumers prefer new materials and experience the aging process of reused products as negative, materials exist that are perceived more positively when showing signs of wear-and-tear [42]. We hence consider it worthwhile to explore how the material choice influences the perception of refurbished personal care products with wear-and-tear.

While prior research established that signs of wear-and-tear and traces of a former user are responsible for these contamination concerns, in this research, we want to explore how to prevent future contamination concerns when designing the first version of a product. By exploring which features of the product evoke contamination concerns, we hope to acquire insights on how to decrease the perceived contamination via the product design. Due to the exploratory nature of this research, we use qualitative research to acquire in-depth knowledge of why product-related factors, personal factors, and context-related factors of personal care products can trigger contamination concerns and how product appearance can help decrease them. We chose to conduct in-depth interviews because such interviews help to reveal rich information about the personal experiences of consumers [43].

## Method

### Procedure and Participants

**Fig. 1:**
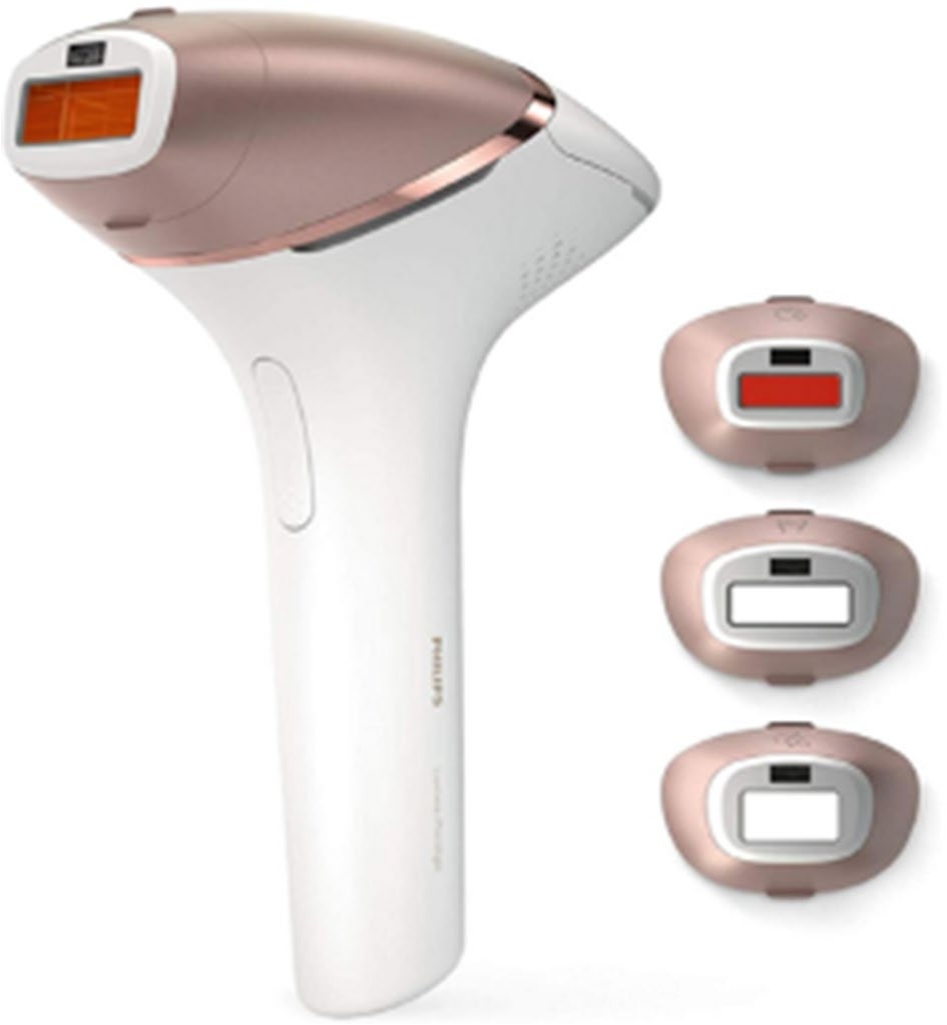
The Philips Lumea Prestige—an intense pulsed light (IPL) device to prevent hair regrowth [48]

Two interviewers conducted audio-recorded semi-structured interviews online with 15 female participants (age: 21 to 67 years, all living in the Netherlands) that had purchased an intense pulsed light device (see Fig. 1).

We decided to interview a total of 15 participants to ensure data saturation. Data saturation is attained when any new interview delivers approximately one or two additional codes. To attain data saturation for homogenous groups, multiple sources propose that a sample size of 10–16 participants is sufficient [44–46]. In our case, data saturation was achieved with 12 interviews, after which each new interview only provided 1–2 new codes. The interviews were conducted online via Zoom due to the Covid-19 pandemic and resulting lockdown between February and June 2021. We interviewed a convenience sample of 15 female participants living in the Netherlands who owned new, second-hand, or refurbished IPL devices to obtain diversity in responses regarding refurbished products (see Table 1). Four participants who had bought a refurbished IPL device were recruited via a banner on the manufacturer’s website. Participants could sign up for this research after they had purchased the device. Five participants with a second-hand IPL device and six participants with a new IPL device were recruited through posts on IPL device Facebook groups, or on the Dutch online second-hand selling platform Marktplaats. The first interview was conducted by interviewer one, but with both interviewers present to synchronize on how to conduct the interviews. All interviews were conducted in Dutch, except for one interview, which was conducted in English.

**Table 1:** Product states of the IPL device that participants owned

Product state	Participant number
New	6, 7, 11, 12, 13, 15
Refurbished	1, 3, 4, 5
Second-hand	2, 8, 9, 10, 14

Participants were first interviewed on their general choice for the IPL device and why they chose it in a new, second-hand, or refurbished state. Furthermore, the product state of the IPL device (e.g., “Can you describe the condition of the product at its arrival?”) and the first product use (“Can you describe your first use experience?”) were discussed to prompt a discussion on contamination concerns for users who had bought a refurbished or second-hand IPL device (hygienic, utility, and territorial contamination concerns [38]) without directly suggesting contamination concerns to participants. For users of a new product, we hoped to spark a conversation on how they perceived the characteristics of the device, such as the laser. Furthermore, to explore the product experience participants had with new, refurbished, or second-hand IPL devices, we discussed how they experienced the product aesthetics (“Can you describe the appearance of the product?”), their emotional experience (“How did it *feel* when you first used the Lumea?”), and why they did (not) choose a refurbished one (e.g., “What made you decide to buy a refurbished device over a new one?”). These questions were based on the framework of product experience [47], which describes different affective responses (aesthetic, emotional, meaning) that can be experienced in human-product interactions. We explored the human-product interaction to prompt a discussion on motivations to choose a product, how the product state (new, second-hand, or refurbished) relates to participants’ choices for the product, and whether there were differences in the interaction with the product. Second, all participants were shown six scenarios that were used to stimulate the imagination of buying a refurbished IPL devices with different attributes and stimulate further thought on refurbished IPL devices with different designs, signs of wear-and-tear, and price. We explained that these products were refurbished, after which participants were asked which one, they would buy if they had been offered this choice and to explain why.

We added these scenarios because consumers’ choice for refurbished products often consists of compromises between different attributes, such as a lower price that comes at the expense of having scratches on the device. We added these scenarios to discuss how participants considered these attributes and which compromises they were willing to make. Interviews lasted between 30 and 40 min. An informed consent was obtained from all individual participants included in the study. Participants received a small compensation (10 euros voucher) for their participation. The study was approved by the Human Research Ethics Committee of Delft University of Technology.

### Stimulus Material—an Intense Pulsed Light (IPL) Device

**Fig. 2:**
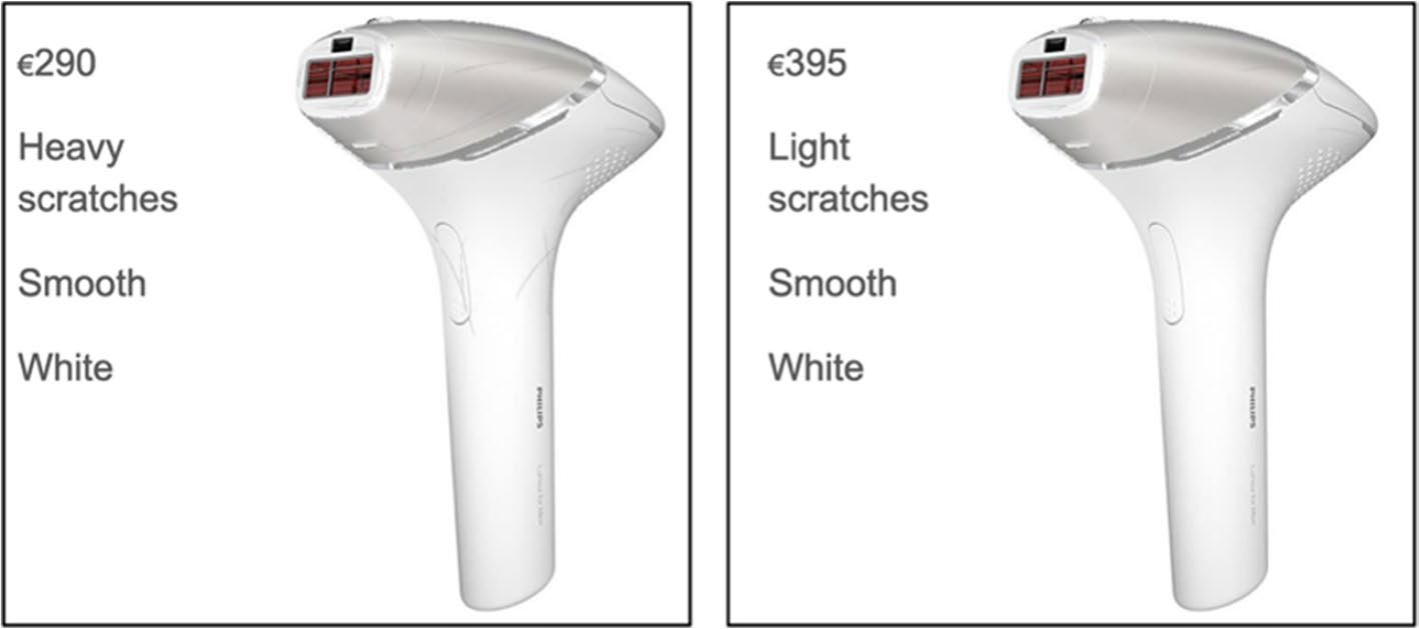
Example scenario with a white IPL device with heavy scratches and lower price (left) and one with light scratches and a higher price (right)

The Philips Lumea Prestige is an IPL device to prevent the regrowth of hair [48]. To use the IPL device, users must first remove their hair (e.g., by shaving), then the IPL device is pressed on the skin (face, legs, armpit, or bikini line), and an IPL flash is activated by pressing on a button. The IPL brings hair follicles into a resting phase and prevents their regrowth. We decided to use an IPL device for our study because it is a personal care product that is intimately used, and therefore more likely to trigger contamination concerns. Furthermore, the price of the device (370–500€) makes it attractive to buy it in a refurbished state because of the considerable amount of money consumers can save. It is designed for a particularly long product lifetime (15 + years) and is available in different product states (new, refurbished, and second-hand). It is therefore a product for which refurbishment makes sense as a lifetime extension strategy, and it was possible to find consumers who bought it in a reused state. The six scenarios that participants were exposed to consisted of two options side by side that differed in color (black or white), material (smooth or rubber), number of scratches (light or heavy), location of scratches (on housing or on buttons or attachment), and price (290–450€). An example scenario is displayed in Fig. 2 and all scenarios are presented in Table 2. The first scenario was used to prompt a discussion on the tradeoff between price and product state (as new vs. with scratches), and the second to evoke a discussion on the material (rubber vs. smooth) in combination with the color of the device. The third scenario was designed to initiate a discussion on the location of signs of wear-and-tear, whereas the fourth scenario was used to spark a discussion on the tradeoff between price and signs of wear-and-tear on functional parts. The fifth scenario was used to elicit discussions on the role of the color of the device and the sixth scenario to trigger a discussion on the tradeoff between price and the amount of wear-and-tear (light vs. heavy). The images were created in Adobe Photoshop.

**Table 2:** Participants were exposed to two IPL devices side by side that differed in state, material, color, and price

Scenario	State	Material	Color	Price
1	As-new state	Smooth	White	395
	Scratches	Smooth	White	290
2	As-new state	Smooth	White	395
	As-new state	Rubber/matt	Black	395
3	Scratches (on buttons and attachment)	Smooth	White	290
	Scratches (on housing)	Smooth	White	290
4	New (not refurbished)	Smooth	White	450
	Scratches (housing, attachment, and buttons)	Smooth	White	290
5	Scratches (housing, attachment, and buttons)	Smooth	White	290
	Scratches (housing, attachment, and buttons)	Smooth	Black	290
6	Heavy scratches (housing, attachment, and buttons)	Smooth	White	290
	Light scratches (housing and attachment)	Smooth	White	395

### Data Processing

All interviews were audio-recorded, transcribed, and analyzed by the principal investigator in Atlas.ti. Codes were developed during two inductive coding rounds. In the first coding round, the first five interviews were analyzed in a collaborative session with the two interviewers. The remaining 10 interviews were analyzed by the principal investigator in a second coding round and resulted in a total number of 232 codes. The first-order codes were categorized into 21 s-order codes that were sorted into 12 themes related to contamination (see the Appendix section for coding tables). Data saturation was reached after 12 interviews, suggesting that our sample size was sufficient.

## Results

Overall, refurbished IPL devices were considered a riskier choice compared to new ones because they were expected to have a reduced product lifetime, to have a lower performance, and to be contaminated by their prior user. In line with prior research [7], participants worried they would make a bad financial investment buying a refurbished IPL device that has an unsatisfactory performance. The main concern was related to utility contamination [38], namely that refurbished IPL devices might have a lower effectiveness in removing hair compared to newer models on the market or of buying a product that simply does not last as long as a new one because of its prior use. These concerns were described to be irrational and mainly based on participants’ gut feelings and prior experiences with refurbished products.

Participant 6 (in response to the question whether she would buy a refurbished IPL if she bought it again): “I think I would still go for a new one. It is more a feeling than that it has a rational reason because I had a refurbished coffee machine in the past and other things. They were not from Philips but another brand, but they broke down very quickly. So, no idea. I would go for a new one again.”

**Fig. 3:**
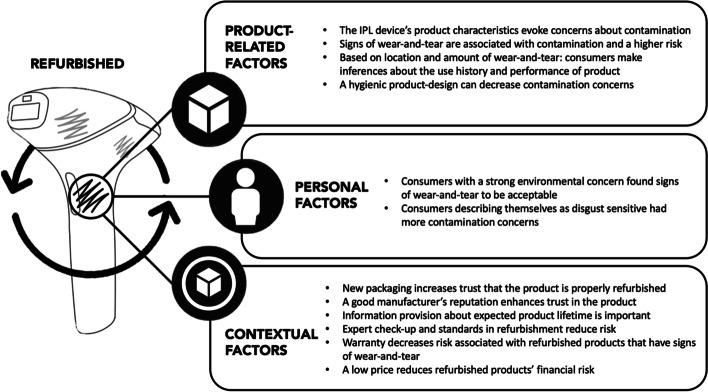
How contamination concerns are related to product-related factors, personal factors, and marketing/context-related factors

In the next sections, we will explain how these contamination concerns are related to product-related factors (e.g., signs of wear-and-tear), personal factors (e.g., disgust sensitivity), and context-related factors (e.g., brand reputation). For an overview of the results, see Fig. 3. An overview of codes and themes can be found in Table 3 (product-related factors), Table 4 (personal factors), and Table 5 (context-related factors) in the Appendix.

**Table 3:** The influence of product-related factors on contamination concerns

Product-related factors	
Themes	Second-order codes
The IPL device’s product characteristics evoke concerns about utility and hygienic contamination	Refurbished IPL devices have a higher performance risk, shorter expected lifetime, and obsolescence risk, which results in a higher financial and time risk
	The IPL device is a scary device because of its laser and fear of it damaging one's skin is increased when it is bought in a reused state
	Refurbished IPL devices have an increased contamination risk
The influence of signs of wear-and-tear on the use history and performance characteristics	Signs of wear-and-tear decrease the expected performance, safety and product lifetime of device
	Based on the amount and location of aesthetic signs of wear-and-tear, users make inferences on what happened to the product (use history)
Signs of wear-and-tear are associated with a hygiene risk	Association of the IPL devices’ prior use with contamination (e.g., particles of a prior user) is strong and makes the IPL device less desirable
	Signs of wear-and-tear make hygiene contamination concerns worse because they make the product harder to clean and evoke associations that the product being marked with traces of a prior user (e.g., smell)
A hygienic product design can decrease contamination concerns	A neat and hygienic product appearance decreases the contamination risk
	The white color of the IPL device is associated with hygiene and this can decrease contamination concerns. Colors on which aesthetic signs of wear-and-tear are not visible also reduce contamination concerns
	Smooth material is favorable for hygiene expectations, rougher material is undesirable because it is harder to clean, more bacteria and dirt stick to it
	Fewer splitlines make the product more hygienic because less dirt can assemble in them

**Table 4:** Consumer characteristics determine the proneness to contamination concerns

Personal factors	
Themes	Second-order codes
Consumers with a strong environmental concern found signs of wear-and-tear to be acceptable	Financial and environmental benefits make signs of wear-and-tear of a refurbished product acceptable (when functionality is guaranteed) to consumers who are concerned about the environment and/or are conscious about money
Consumers with a high disgust sensitivity had more contamination concerns	Reused IPLS need to be cleaned before first use because consumer is disgust sensitive

**Table 5:** The influence of marketing/context-related factors on contamination concerns

Contextual factors	
Themes	Second-order codes
A low price can reduce financial risk associated with the product and enhance the financial benefit	A low price can incentivize consumers to buy a refurbished Lumea with heavy wear-and-tear when the functionality is guaranteed (but the price difference needs to be large enough)
Warranty decreases risk associated with refurbished products that show signs of wear-and-tear	Warranty decreases the risk associated with refurbished products because one knows whom to contact if the product does not work and this determines the product choice
	Warranty can make refurbished products with signs of wear-and-tear more desirable because it compensates for the perceived risk that the device is dysfunctional or has a lower expected lifetime
Expert check-up and standards in refurbishment reduce risk that product is unclean or malfunctions	The fact that a refurbished product is checked by an expert and therefore adheres to a certain standard makes them a safer choice compared to second-hand products
	Standards in refurbishment make refurbished products less risky
Information provision about expected product lifetime and prior use	It is important to know for how long the refurbished product is conceptualized to last and how long it has been used
Image of manufacturer and refurbisher	The trustworthiness and good reputation of a manufacturer has a positive influence on the choice for refurbished/reused products because it enhances trust in the quality of a product and the refurbishment process
New packaging	New packaging indicates that the product has been processed and refurbished

### The Influence of Product-Related Factors on Contamination Concerns

An IPL device is a “scary” device because of its product features, such as the light flashes. The IPL is considered to be an innovative device and its technology is not well understood by consumers. Some participants associated IPL treatment with similar technologies, which are painful when being administered, such as laser hair removal used at professional beauty salons. It is therefore scary to use the IPL device for the first time and even scarier to use it in a refurbished state because the risk is higher that it may malfunction. It is therefore prone to trigger concerns about utility contamination, the belief that a product’s functionality is impacted because of its prior use [38].

Participant 14: “It is a technical product that I don’t fully understand, and I wouldn’t know if it properly functions. That was a concern like: is it [the refurbished IPL device) still properly functioning and safe? It didn’t prevent me from trying an old one, but the thought was there. (…) It is and stays a laser. It just doesn’t give you a good feeling. And we know about laser that if you don’t use it correctly, or if the product is not put together correctly, that could perhaps be dangerous.”

Additionally, the IPL device is intimately used. When using the IPL device, participants have to press it on their skin on areas that they want to be hairless, such as the armpits or bikini line. This makes it more vulnerable to hygienic contamination [38] when it is bought in a refurbished or second-hand state because users are concerned about finding traces of prior users on the device.

Participant 2 (owner of a second-hand IPL device): “I know that the Lumea doesn’t remove hair immediately, but I was cautiously looking if there would be something here from somebody else. Yeah, but there wasn’t. Specifically, I removed the head (attachment), and I was looking if there would be black marks here or if there would be anything in the splitlines on the side.”

Refurbished IPL devices were inspected for signs of use or physical traces, but not cleaned before the first use because they were expected to be clean.

Participant 5 (owner of a refurbished IPL device): “No, no – I did not clean it before I used it for the first time. I trusted that it is clean.”

Comparably, second-hand IPL devices were inspected for particles of a prior user (e.g., skin particles, hairs and smells) and cleaned thoroughly before the first use.

Participant 2: “It was clean when I got it. It’s more like: Somebody owned it before, so let's clean it! Even though it was already clean. I looked at it and I couldn’t see any signs of use in a way, yeah. I read in the instruction manual that, after it has been used for a while, it can be that there are some speckles that show up on the light part. Yeah, I did not see any speckles. Although there was one really tiny one, but not that it would look like it had been extensively used.”

#### Signs of Wear-and-Tear Increase the Contamination Risk

Signs of wear-and-tear increased the risk of contamination. Some participants stated that signs of wear-and-tear were acceptable on refurbished IPL devices as long as they can still be cleaned well.

Participant 3: “I would go for the Lumea with wear-and-tear. I absolutely think scratches, yes, they come with refurbished products, given that it can still be cleaned well, with detergent.”

Moreover, some participants would choose a more expensive IPL device that is new or has no signs of wear-and-tear because of the negative association with its prior use, and subsequently, the feeling that it is contaminated. The signs of use were not only associated with skin particles of previous owners (hygienic contamination; [38]) but also indicated by the smell of a product (territorial contamination; [38]) which make refurbished products a less attractive option.

Participant 12: “I would choose a more expensive Lumea (without signs of wear-and-tear) because I find that the other one has too strong associations with being used, that it was used by somebody else. And also, I am very sensitive to smells, to say. It is just like a book that has been lying around at somebody else’s smelly house. It goes into the paper. So, I am afraid of the smell and remainders that are still on it.”

The presence of signs of wear-and-tear was perceived to decrease the performance, safety, and expected product lifetime of the IPL device. For some participants, a warranty was sufficient to guarantee that the refurbished IPL device is safe and fully functional. Other participants, however, felt that the IPL device is safer without signs of wear-and-tear, acknowledging that this belief might be irrational.

Participant 14: “Well, I know that (the refurbished IPL device) went through a check-up, so the scratches should actually not mean anything in terms of safety and functionality of the product. But I think that I would go for this one (IPL device without scratches) because for me as a person, the scratches would give me an irrational feeling of anxiety. And then I think it is not worth it.”

Concerns about the product’s safety and potential dysfunctionality were influenced by the amount and location of wear-and tear. Participants believed that the more signs of wear-and-tear were present, the riskier it was to buy the refurbished IPL device because of two reasons:

First, participants made inferences on how the previous owner handled the IPL device based on the heaviness of the signs of wear-and-tear. While light signs of wear-and-tear reflect normal use, heavy signs of wear-and-tear reflect bad treatment. For example, it is hard to scratch the IPL device according to participants’ experiences, heavy signs of wear-and-tear give the indication that the product has been dropped multiple times, and hence the risk is higher that technical parts are damaged.

Participant 10: “Looking at the picture, I see that there are scratches literally everywhere. That thing has fallen from the stairs, fell on the floor and the dog chewed on it. Let’s say, I know first that it has been used a lot and, second of all, that it was treated badly.”

Second, heavy signs of wear-and-tear were associated with a longer use time and hence indicate a reduced product lifetime.

Participant 10: “That (heavy scratches on the IPL device) doesn’t help very much with the life cycles of the thing. They (IPL devices) only have a limited number of flashes, that are only a number. So, if I look at the IPL device (with heavy-signs of wear-and-tear) then I think that you are through half of your light flashes. That is not handy.”

Furthermore, participants made inferences about how signs of wear-and-tear were made based on their location. Scratches on the body of the product signaled normal signs of use. Scratches on parts that touch the skin, parts that are hard to damage, or functional parts, such as the buttons or the attachments, indicate bad treatment by the previous user and an increased functionality risk.

Participant 14: “I think to me, scratches on the attachment (product part that is pressed on skin) would make a difference. Even if they are just on the plastic part and not on the window, let's say. I know myself that this is irrational, but sorry. I would be afraid that it (the attachment) can break, and the laser comes through places where it shouldn’t be.”

Moreover, participants indicated that the device might have a lower performance because the outside state of the IPL device reflects its inside.

Participant 4: “I think because the buttons have been scratched, I think that there could be something going wrong on the inside as well and through this that the button is harder to press in.”

#### A Hygienic Product Design Can Decrease Contamination Concerns

A hygienic product design can help decrease contamination concerns. We define a hygienic product design as a product design that is easy to clean and in turn that enables to see how clean the product is. Overall, the IPL device was rated to have a hygienic design and that made the device more desirable in a refurbished state.

Participant 7: “Yes, it’s good if it is designed in such a way that it can just work hygienically. Of course, its (the device’s) hygiene also depends on the person in question: on how their skin was treated and how it (the device) was treated.”

A neat and hygienic product appearance can decrease the feeling of contamination by being cleanable and by designing the product in a way that the visibility of wear-and-tear is minimized. This is in line with prior research indicating that participants prefer refurbished products without signs of wear-and-tear because if contamination is not in sight, it is not in the mind [13].

Participant 14: “It’s especially important that the Lumea is clean, isn't it? But still, it just gives a nice feeling when it also looks a bit neat. I would go for one where the scratches are less visible because I know about myself that if I am using it (the one with fewer scratches), then I wouldn’t pay attention to it and that would do something with my feeling of safety because the scratches are just a little bit less visible.”

A hygienic appearance was associated with the texture of the IPL device, the color, and the product form. The white color of the IPL device evoked association with hygiene and being easy to clean.

Participant 13: “Then give me the white one anyway. And yes, I think because of hygiene considerations anyway. I think this is easier to clean. It is nonsense, of course because it's the same material (as the black one), so it doesn't make sense.”

On the other hand, some participants preferred a black IPL device because the scratches were less visible on it.

Participant 13: “Maybe I would go for the black one because you can see the scratches less.”

Next to the color, the material was critical when it comes to hygiene. Participants favored a smooth texture because it is easier to clean and participants could check whether the IPL was clean when they received it.

Participant 2: “It’s very hygienic (the material) because it’s very smooth. … if it was a coarse material then maybe you know certain skin residue or bacteria, or something might get stuck in it. But in this way, it just yeah, it seems like something that you might be able to clean.”

Additionally, participants appreciated it if there were few splitlines and holes in the object in which dirt could assemble, even though they understood that these are necessary to make a product that can be disassembled.

Participant 2: “I would like it if there were less of the splits (splitlines), but probably yeah, if you want a product that can be refurbished that it needs to have this so that we can reach the inside and remove and exchange components.”

### Consumer Characteristics Determine Consumers’ Proneness to Contamination Concerns

Consumer characteristics, such as pro-environmental concern, can determine how people perceive and judge the presence of signs of wear-and-tear [15]. Participants who had bought refurbished IPLs were environmentally motivated and were also more willing to accept signs of wear-and-tear given that the product was checked by the manufacturer and therefore fully functional. This adds to previous research pointing out that a high environmental concern is associated with a willingness to choose a refurbished product with signs of wear-and-tear [13].

Participant 1: “Yes, yes, I am really very conscious about sustainability, so I would go for the one with scratches. Because I know, I read that the manufacturer is going to check them.”

On the other hand, participants who said about themselves that they were sensitive to feeling disgusted would typically be more concerned about the contamination risk of the IPL device and therefore rather buy a new one. This is in line with prior research showing that there are individual differences between consumers in the frequency and degree to which they feel disgusted (disgust sensitivity; [49]). Furthermore, this finding extends prior research demonstrating disgust sensitivity as a predictor of avoidant behavior towards stimuli that are deemed to be disgusting, such as recycled water [21].

Participant 14: “It was a little bit of a struggle because I didn’t know how to clean the device. I couldn’t find it back in the manual because you naturally don’t want to break the window (glass part of the attachment). And you’re only allowed to clean it with a little bit of water and a moist cloth if I understood correctly. And well, maybe I am also just sort of a disgust sensitive person and then again, I think about: Is it completely clean?”

Personality characteristics set a baseline of how contamination of personal care products is perceived [13]; while one should not attempt to change consumers’ characteristics, appropriate marketing can help to ease their perceived contamination concerns.

### Marketing Strategies Can Compensate for the Contamination Risk

While contextual factors cannot prevent contamination risks, they can help to make refurbished products more attractive through compensating for the contamination risk by enhancing the financial and environmental benefits [30, 50] and decrease the perceived financial and performance risk [7].

First, refurbished IPL devices are an economic option because consumers can save 100–200 euros when buying a refurbished or second-hand one. The higher the price difference between a new IPL device and a refurbished one, the lower the risk that one makes a bad financial investment. If the price is low enough, participants were therefore more inclined to choose a refurbished IPL device with signs of wear-and-tear.

Participant 13: “I think the price difference with this one (IPL device with heavy scratches) is worth thinking about buying one with scratches.”

Confirming prior research [10, 15], we found that the warranty and an expert check-up during the refurbishment process can make the product less risky and incentivize consumers to choose for a refurbished IPL device. The fact that a refurbished product is checked by an expert and therefore adheres to a certain standard makes them a safer choice compared to second-hand ones. A try-and-buy period and an extended warranty reduce the risk because they know whom to contact if the product does not work and that they will receive another functional product in that case.

Participant 15: “Indeed, there is a real warranty, you know what you are buying. There is a number you can call when there is something not right. So, those are all things that make me feel: okay, I would consider buying that there (participant points towards refurbished IPL device in the presented dilemmas).”

A good manufacturer’s reputation [7, 17, 32] is influential because it increases the perceived quality of the product, whether it can be refurbished well and whether the manufacturer can be trusted to grant the promised warranty. Additionally, the reputation of the refurbisher and, in our case, also the manufacturer, influenced whether people trust that the product is fully functional and safe to use. The reputation was determined by a good brand image and reviews.

Participant 3: “I just have quite a lot of faith in Philips in terms of factory or equipment. And of course, I know people around me who have also used Philips and they are very satisfied with it. But I think I would give others (other brands) a chance too, but you know, Philips just has a good name.”

The retail experience also has an influence on how desirable and risky refurbished products are. One concern that consumers had been is that the product was sold as refurbished but was not processed properly and therefore might not have been tested, cleaned, and repackaged. New packaging indicates that the product has been processed and properly refurbished (tested, checked, cleaned) and this can decrease contamination concerns.

Participant 14: “I trust a brand like Philips, that stands behind it and promises me that if they put a whole new package on it and sealed it and checked it and say: we cleaned it, that they will have really cleaned it.”

Finally, the information provision about the refurbished product determines the IPL device’s desirability. Knowing how long a refurbished product is designed to last and how long it has already been used would help the consumer to determine whether the product is a good investment.

Participant 13: “I would question: how long is it (the refurbished IPL) going to last? You know, if they would say that it can be used for a certain number of flashes and it would last for that long. Okay, they don’t know how many flashes you use per time but it would make a difference.”

## Discussion and Conclusions

This research contributes to the literature by explaining why consumers’ contamination concerns increase the perceived riskiness of refurbished products and how they can be reduced. To keep refurbished personal care products at their highest value, we propose five design strategies to minimize contamination concerns by designing a product that looks and smells hygienic even after multiple lifecycles. Through this, we hope to address not only rational concerns that consumers have but also emotional ones. For refurbished personal care products with signs of wear-and-tear that cannot be eliminated, we suggest marketing strategies that can mitigate consumers’ contamination concerns and therefore enhance their desirability.

Our findings demonstrated that consumers generally deemed refurbished personal care products with signs of wear-and-tear to be a riskier choice. Confirming prior research [7, 15, 38], the risk that the device malfunctions was expected to be higher, participants estimated that products would have a shorter product lifetime (utility contamination) and would be contaminated by a prior user (hygienic and territorial contamination). These risks were increased for refurbished IPL devices with heavy signs of wear-and-tear and signs of wear-and-tear on functional components, which is in line with prior research stating that aesthetic signs of wear-and-tear signal contamination by a former user [38]. We additionally argue that the aesthetic wear-and-tear may signal to consumers that the functionality risk is higher, and the device is more likely to be contaminated with traces of a prior user. Building on prior research [38], we found that based on the location and amount of wear-and-tear, users make inferences on how the prior user has treated the device which in turn indicates a lower functionality of refurbished personal care products.

### Design Strategies for Personal Care Products

When designing products for multiple lifecycles, it is important to understand that contamination concerns are more feelings than rational thoughts. Even though consumers rationally know that products are tested, repaired, and cleaned during the refurbishment process, feelings of unease due to contamination persist. When designing for refurbishment, designers therefore need to address feelings in addition to cognitions. We therefore recommend designing products in a manner that minimizes the feeling of contamination by reducing signs of wear-and-tear and evoking associations that make the consumer feel safe. Prior research suggested designers use materials that are either aesthetically durable and keep an as-new appearance over time, or by using materials that can be easily returned into an as-new state [13]. Wallner et al. [13] recommended using materials that can be easily sanded off (e.g., stainless steel), self-healing materials [51, 52], or to use coatings that can easily be renewed. Based on our findings, we would like to extend existing design strategies for refurbishment by recommending the following strategies specific to personal care products:

1. Evoking associations with hygiene: The white color in our study was associated with hygiene. While this was acknowledged to be an irrational association, white products were perceived to be cleaner than black ones. We therefore recommend testing which colors are associated with cleanliness and therefore help reduce consumers’ contamination concerns.
2. Making signs of wear-and-tear less visible: Colors that made signs of wear-and-tear less visible were considered desirable for refurbished personal care products. Especially for devices that will be heavily used during their life cycle, we recommend using coloring, patterns, or textures that help to minimize the visibility of wear-and-tear because if signs of wear-and-tear are “out of one’s sight,” indeed, they are “out of one’s mind.”
3. Smooth materials: For hygienic products, we recommend using materials that are smooth over materials that are rougher as consumers have the association that smooth materials are easier to clean while in rougher materials, dirt, and bacteria assemble.
4. Few split lines: Similarly, consumers preferred as little split lines in hygienic products as possible so that no dirt can assemble in them.
5. A clean product smell: Personal care products with wear-and-tear were associated with a bad smell. To counter this perception, we therefore recommend not only addressing contamination concerns aesthetically but through all senses by giving the product an as-new or clean smell.

### Marketing Strategies to Compensate for Contamination

While marketing strategies cannot prevent contamination concerns of products with signs of wear-and-tear, they can make refurbished products a less risky choice despite being contaminated. By offering refurbished products for an economical price, and with an extended warranty, retailers can reduce the risk consumers experience [7, 17]. Furthermore, to foster trust that the product was processed and fully functional, we recommend communicating that the product was checked by an expert, by offering it in new packaging and fostering a good brand image. Additionally, retailers could decrease the risk that the product does not last as long as a new product by being transparent about how long the product has been used for and for how long it is conceptualized to last. This could be achieved through providing a product lifetime label that gives consumers an estimation for how long they could use the product [53]. To keep products at their highest economic and functional value, contamination should, however, be considered when designing a personal care product.

### Limitations and Future Research Directions

While all our participants owned an IPL device, some of them did not have the actual experience of receiving and using a refurbished IPL device but owned a new or second-hand one. A limitation of our study is that we used pictorial stimuli to evoke associations with contamination for refurbished personal care products. We realize that showing signs of wear-and-tear in a pictorial representation of a product differs from seeing such signs of wear-and-tear in real life. For example, signs of wear-and-tear were portrayed in our stimuli to be quite severe. Realistic signs of wear-and-tear would often be less extensive, which may also result in less negative reactions. We nevertheless decided to manipulate it in this way because more subtle signs of wear-and-tear would have been difficult to see in the scenarios that we shared with participants online. Furthermore, we recognize that contamination addresses more senses than just the visual. We therefore believe that it would be interesting for future research to validate our findings in an in vivo setting by testing contamination concerns of refurbished products with physical products demonstrating actual signs of wear-and-tear.

Additionally, we only discussed participants’ first use experiences and expectations of buying a refurbished or second-hand IPL device. It would be interesting to test in future research whether contamination concerns are long-lasting due to visible signs of wear-and-tear or decrease after the participant has used the product a few times.

Another limitation of our research is that we focused solely on IPL devices as stimuli. This could have influenced our findings in various ways: first, compared to other refurbished products, the IPL device was manufactured, sold, refurbished, and resold by the same party, which is the manufacturer. Future research could test whether this had an effect compared to products that are refurbished and resold by a different reseller; consumers may perceive more risk in the refurbishment process due to the involvement of a third party. Second, the device we tested is a luxurious, and therefore expensive, personal care product. The financial benefit of buying a refurbished IPL device is therefore higher than for other, less expensive products. For other personal care products, such as electric toothbrushes or shavers, refurbishment might be less financially attractive and evoke even more contamination concerns in consumers [11]. For these product categories, other strategies should be tested, such as emphasizing that those parts touching the skin have been replaced during the refurbishment process. Fourth, due to the nature of the device, we only interviewed female participants who can afford a luxurious IPL device; gender differences have shown to influence decision-making about purchasing technological devices [54]. Future research should therefore explore more diverse consumer responses to refurbishment for more product categories sensitive to contamination to validate and extend our suggested design strategies. Furthermore, while this sample size was sufficient to give a comprehensive overview of the experiences of a homogenous sample with females using IPL devices, future research should validate our findings with larger and more diverse samples including participants of different age ranges, genders, and ethnicities.

### Conclusions

This research sought to understand why consumers’ contamination concerns increase the perceived riskiness of refurbished products and how they can be reduced. Our data suggests that consumers perceive refurbished personal care products with signs of wear-and-tear to be a riskier choice and expected that the device would malfunction, have a shorter product lifetime, and would be contaminated due to the previous use. To keep refurbished personal care products at their highest value, we therefore propose design strategies to prevent contamination concerns by designing a product that smells and looks hygienic after multiple lifecycles. For refurbished personal care products with signs of wear-and-tear that cannot be eliminated, we propose mitigating consumers’ contamination concerns with marketing strategies. Contamination has shown to be a barrier in consumer adoption of various circular products beyond refurbishment, such as clothing in product service systems [25], remanufactured consumer electronics [9], or reusable food packaging [26, 27], and is therefore important to address in research. This work contributes to the literature on user-centric design and marketing of refurbished personal care products, and the understanding of the potential of strategies to reduce contamination concerns in the circular economy more broadly. Finally, manufacturers could use these insights to design products that retain their value over multiple life cycles and establish a promising market strategy for refurbished personal care products, which is both commercially relevant and creates a substantial environmental benefit.

## Data Availability

The transcripts are published in the 4TU research data repository https://doi.org/10.4121/19375001.
